# Long-term effects of bosentan on quality of life, survival, safety and tolerability in pulmonary arterial hypertension related to connective tissue diseases

**DOI:** 10.1136/ard.2007.079921

**Published:** 2007-11-30

**Authors:** C P Denton, J E Pope, H-H Peter, A Gabrielli, A Boonstra, F H J van den Hoogen, G Riemekasten, S De Vita, A Morganti, M Dölberg, O Berkani, L Guillevin, (on behalf of the TRacleer Use in PAH associated with Scleroderma and Connective Tissue Diseases (TRUST) Investigators)

**Affiliations:** 1 Royal Free Hospital, London, UK; 2 St Joseph’s Healthcare, London, Ontario, Canada; 3 Med. Universitätsklinik Freiburg, Freiburg, Germany; 4 Azienda Ospedaliera Umberto I, Ancona, Italy; 5 Vrije Universteit Medisch Centrum, Amsterdam, The Netherlands; 6 Universitair Medisch Centrum St. Radboud, Nijmegen, The Netherlands; 7 Charité Universitätsmedizin, Berlin, Germany; 8 Rheumatology Clinic, DPMSC, Azienda Ospedaliero Universitaria, Udine, Italy; 9 Actelion Pharmaceuticals Ltd., Allschwil, Switzerland; 10 Hopital Cochin, Paris, France

## Abstract

**Objectives:**

This study investigated the long-term effects of bosentan, an oral endothelin ET_A_/ET_B_ receptor antagonist, in patients with pulmonary arterial hypertension (PAH) exclusively related to connective tissue diseases (CTD).

**Methods:**

A total of 53 patients with PAH related to connective tissue diseases (PAH–CTD) in World Health Organization (WHO) functional class III received bosentan 62.5 mg twice a day for 4 weeks and then 125 mg twice a day for 44 weeks in this open non-comparative study. Assessments at weeks 16 and 48 included WHO class, clinical worsening, quality of life (Short-Form Health Survey (SF-36) and health assessment questionnaire (HAQ) modified for scleroderma), and survival (week 48 only). Safety and tolerability were monitored throughout the study.

**Results:**

At week 48, WHO class improved in 27% of patients (95% CI 16–42%) and worsened in 16% (95% CI 7–29%). Kaplan–Meier estimates were 68% (95% CI 55–82%) for absence of clinical worsening and 92% (95% CI 85–100%) for survival. Overall changes in quality of life were minimal. There were no unexpected side effects observed during the study.

**Conclusions:**

In most patients, bosentan was associated with improvement or stability of clinical status. The 92% estimate for survival at 48 weeks is a significant achievement in this patient population.

Pulmonary arterial hypertension (PAH) is a progressive and often fatal complication of connective tissue diseases (CTDs) such as systemic sclerosis (SSc), systemic lupus erythaematosus (SLE), and overlap or mixed connective tissue disease (MCTD).^1^^–^3 CTDs are disorders characterised by a wide range of vascular, inflammatory, and fibrotic manifestations in many organs including lung, kidney, and skin. Over the past decade, advances in medical treatment have improved the management of the complications associated with CTDs. Patients with SLE have benefited from immunosuppressive treatments,^4^ while improved management of the specific complications associated with SSc and MCTD (eg, scleroderma renal crisis), has improved prognosis.^5^ However, PAH remains a major cause of long-term morbidity and mortality. The reported symptomatic PAH prevalence measured by right heart catheterisation is 8–12% in patients with SSc,^1^ ^6^ 6–11% in patients with SLE,^7^ ^8^ and up to 10–45% in patients with MCTD^3^ as measured by echocardiography and/or right heart catheterisation.

In these patients, early detection of PAH and a multidisciplinary approach to diagnosis and treatment in specialised PAH and/or CTD centres may improve clinical outcome.^9^ ^10^ Therapeutic approaches for PAH–CTD are based on those used for treating idiopathic PAH (iPAH).^11^ Anticoagulation, diuretics, and oxygen supplementation are often used although the benefit of this supportive therapy has not been demonstrated in PAH–CTD.^1^ Prostacyclin analogues may improve exercise capacity and pulmonary haemodynamics in these patients.^12^^–^15 However, despite treatment, patients with PAH–CTDs are functionally impaired with a decreased health status and a poor prognosis. In the absence of concomitant PAH, survival of patients with SSc exceeds 90% at 1 year^16^ ^17^ but once PAH has been diagnosed, it decreases to 50%,^18^ ^19^ which is worse than for patients with iPAH (84%).^19^ The risk of death from PAH related to SSc is threefold higher than from iPAH.^19^

Bosentan is an oral dual (ET_A_ and ET_B_) endothelin-1 receptor antagonist. In placebo-controlled clinical trials and in long-term extension studies,^20^^–^22 bosentan was well tolerated, improved exercise capacity and haemodynamics, and delayed the time to clinical worsening in patients with iPAH and PAH–CTD. Survival estimates at 1 and 2 years were 86% and 73%, respectively, in a subgroup analysis of patients with PAH–CTD.^23^ Improvement in quality of life (SF-36 instrument) has been reported after 3 and 6 months of bosentan treatment in patients with iPAH and PAH–CTD (59% and 41%, respectively) participating in the VITAL study.^24^ However, changes in health-related quality of life have not been assessed together with survival. Since the concomitant assessment of these two aspects is critical to appreciate overall outcome, the present multi-centre European study was designed to investigate changes in health-related quality of life together with survival over a 48-week observation period in patients with PAH exclusively related to CTD.

## PATIENTS AND METHODS

### Patients

Included patients (over 18 years of age) had PAH in World Health Organization (WHO) functional class III^25^ related to diffuse or limited SSc, MCTD, or SLE (other CTDs were excluded). PAH was confirmed in all patients by right heart catheterisation requiring mean pulmonary artery pressure ⩾25 mmHg at rest, pulmonary vascular resistance >3 Wood units, and pulmonary capillary wedge pressure <15 mmHg.^26^ This catheterisation was performed within 6 months prior to the start of bosentan therapy. Signs of right heart failure, if present at baseline, were required to be stable and patients were required to have received adequate diuretics treatment prior to bosentan initiation. Total lung capacity (TLC) was required to be above 50% of predicted, to exclude patients with severe interstitial lung disease. Patients were also excluded if they had received any PAH treatments (except anticoagulants) within 1 month of screening, if they were receiving or were expected to receive epoprostrenol or prostacyclin analogues for more than 2 consecutive weeks, or if they had received glibenclamide, cyclosporin A, or tacrolimus within 1 week of screening. Selective phosphodiesterase inhibitors and endothelin receptor antagonists other than bosentan were not allowed during the study. Disease-modifying antirheumatic drugs (DMARDs) were allowed provided the patient had been stable on treatment for 3 months prior to bosentan initiation.

The study was conducted according to the most recent amendments to the Declaration of Helsinki, and in adherence to Good Clinical Practice guidelines. Local institutional review boards or independent ethics committees approved the protocol. Written informed consent was obtained from all patients.

### Study design and procedures

The study was a prospective single-arm trial and was conducted in 23 centres in 8 European countries. Patients received bosentan 62.5 mg twice a day for 4 weeks followed by the 125 mg twice a day target dose for 44 weeks, in addition to stable antirheumatic treatment. Patients who did not tolerate the 125 mg twice a day target dose were down titrated to the starting dose.

Patients were evaluated on an outpatient basis at baseline and at weeks 4, 8, 16, 24, 36, and 48 or at premature withdrawal. Efficacy assessments included the change from baseline to weeks 16 and 48 in WHO functional class, the time from baseline to clinical worsening (defined as the combined endpoint of death or hospitalisation due to PAH complications, use of epoprostenol or prostacyclin analogues for worsening of PAH, lung transplantation, discontinuation due to worsening of PAH), and the time to death.

Health-related quality of life and disability were evaluated at baseline and week 48, with a generic instrument (the Medical Outcomes Study 36-Item Short-Form Health Survey (SF-36))^27^ and a disease-specific instrument (the scleroderma modified Health Assessment Questionnaire (HAQ)).^28^ Both instruments have been validated in a variety of chronic diseases including SSc^28^^–^32 and SLE.^33^ ^34^

In the SF-36 questionnaire, 35 items cover 8 domains of health: physical functioning, role limitation caused by physical functioning, bodily pain, general health perceptions, vitality, social functioning, role limitation caused by emotional problems, and mental health. The patient’s responses are first reported on a scale from 0 to 100 (higher score indicates better health-related quality of life) for each domain following item weighing and additive scaling, these eight scale scores (0–100) are finally transformed (using a linear z-score transformation) to correspond to a mean of 50 and standard deviation of 10 in the 1998 general US population (norm-based scale scores). A 36th item (health transition) asks respondents about any health changes over the past year using five assessment categories, “much better” (category 1), “somewhat better” (category 2), “about the same” (category 3), “somewhat worse” (category 4), and “much worse” (category 5).

The HAQ assessment includes 20 questions in 8 domains of functional activities: dressing, rising, eating, walking, hygiene, reach, grip, and usual activities. The patient’s responses are reported on an ordinal scale from 0 (no disability) to 3 (complete disability). The HAQ disability index is the average score across the eight domains. In addition, six visual analogue scales (VAS) evaluate disease-specific organ system symptoms: pain, digital ulcer, gastrointestinal, vascular, and pulmonary involvement, and overall disease severity, with scores standardised to a continuous scale from 0 (no symptoms) to 3 (worst symptoms).

Safety was assessed by the reporting of adverse events up to 1 day after study drug discontinuation and serious adverse events up to 28 days after study drug discontinuation. Liver function tests were performed at monthly intervals. Any marked laboratory abnormality was reported as an adverse event.

### Statistical methods

Sample size selection was empirical for this open, single arm study. Statistical analyses were performed in an exploratory fashion. For numerical endpoints, the change from baseline is presented with 95% two-sided confidence intervals, based on asymptotic normality assumption. For dichotomous endpoints, the proportion of patients is provided with 95% two-sided CI, based on the binomial exact distribution. For time-to-event endpoints, Kaplan–Meier estimates are presented with 95% two-sided CI calculated from Greenwood’s formula. All treated patients were used for the survival analysis.

Values for missing assessments of WHO functional class, SF-36, and HAQ/VAS were derived by carrying forward the last observed post-baseline assessment. For patients who died, underwent lung transplantation, or discontinued study medication due to worsening of PAH prior to the considered timepoint (week 16 or week 48), the most conservative approach was used in case of missing assessment. The missing assessment was replaced with the worst value out of: (1) the last available post-baseline value observed for this patient, or (2) the worst value observed at the considered timepoint (ie, week 16 or week 48) over all other patients.

## RESULTS

A total of 53 patients were treated over a 1-year period and received bosentan from July 2003 to August 2005. The median exposure to bosentan was 48.6 weeks (range 0.7 to 56.7 weeks). During the study, 17 patients (32%) were prematurely discontinued from study treatment because of an adverse event (n = 14, 26%), sudden death (n = 1, 2%), disease progression (n = 1, 2%), or loss to follow-up (n = 1, 2%). In total, 36 patients out of 53 completed the study.

### Patient demographics and disease characteristics

Patient demographics and disease characteristics are presented in table 1. The majority of patients were female and the mean (SD) age was 63 (13) years. Of 53 patients, 42 had SSc, 29 patients had limited and 13 patients had diffuse SSc. There were six patients with MCTD and five with SLE. Twelve patients had a history or evidence of lung fibrosis at baseline. In all but one of these cases was FVC greater than 60% of predicted.

**Table 1:** Demographics and patient characteristics at baseline

Parameter	Value
Male/female, n (%)	9 (17%)/44 (83%)
Age, years (mean (SD))	63 (13) (range 22–79)
Weight, kg (mean (SD))	67 (13) (range 40–99)
Caucasian/Asian	51 (96%)/2 (4%)
Aetiology, n (%):	
Limited systemic sclerosis	29 (55%)
Diffuse systemic sclerosis	13 (25%)
Mixed connective tissue disease	6 (11%)
Systemic lupus erythaematosus	5 (9%)
Time from CTD diagnosis, weeks (mean (SD))	431 (503) (range 0–2227)
Time from PAH diagnosis, weeks (mean (SD))	45 (66) (range 1–236)
Signs of right heart failure, n (%)	8 (15%)
Patients with at least one digital ulcer, n (%) (n = 50)	15 (30%)
Right heart catheterisation:	
Mean pulmonary arterial pressure, mmHg (mean (SD))	39.5 (12.6)
Cardiac index, litres/min/m^2^ (mean (SD); n = 49)	2.9 (0.9)
Mean pulmonary capillary wedge pressure, mmHg (mean (SD); n = 51)	10.1 (4.4)
Pulmonary vascular resistance, dyn/s/cm^–5^ (mean (SD); n = 47)	559.4 (371.5)
Total lung capacity, % (mean (SD); n = 46)	80.8 (18.3)
Forced vital capacity, % of predicted (mean (SD))	85.9 (23.6)
Concomitant treatment, n (%):	
Antithrombotic agents*	41 (77%)
Antacids/drug for treatment of peptic ulcer and flatulence	40 (75%)
Calcium channel blockers	32 (60%)
Corticosteroids†	30 (57%)
Diuretics	28 (53%)

Unless otherwise stated, n = 53.

*Mostly acenocoumarol, warfarin, or acetylsalicylic acid. †Mostly prednisolone.

CTD, connective tissue disease; PAH, pulmonary arterial hypertension.

### Concomitant corticosteroid therapy

The numbers (percentages) of patients treated with prednisone or prednisolone included: 7 (54%) patients with diffuse SSc (mean dose: 12.4 mg; range: 5–40 mg); 12 (41%) patients with limited SSc (mean dose: 10.0 mg; range: 2.5–40 mg); 2 (40%) patients with SLE (mean dose: 11.3 mg, range: 5–30 mg); and 6 (100%) patients with MCTD (mean dose: 9.1 mg, range: 2.5–25 mg).

### WHO functional class

**Figure 1:**
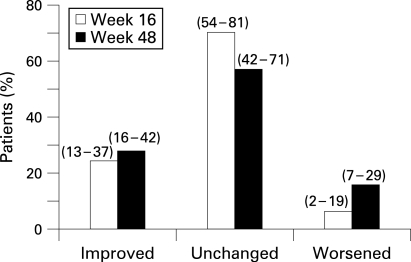
Improvement/worsening in World Health Organization (WHO) functional class at week 16 and week 48. Two patients at week 16 and three at week 48 were discontinued because of pulmonary arterial hypertension (PAH) worsening/death and were assigned WHO functional class IV at week 16 and week 48, respectively, as per protocol. (n = 51). Confidence intervals (95%) are indicated in brackets.

All patients were in WHO functional class III at baseline. At week 16, WHO class improved in 12 out of 51 patients (24%), remained stable in 35 patients (69%), and worsened in 4 patients (8%). At week 48, it improved in 14 out of 51 patients (27%), remained stable in 29 patients (57%), and worsened in 8 patients (16%) (fig 1).

### Clinical worsening

**Figure 2:**
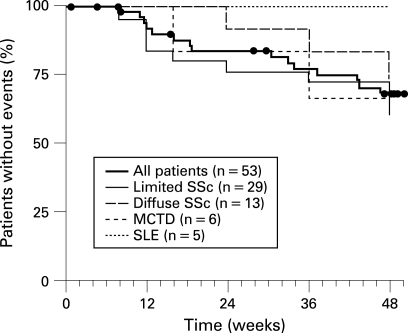
Kaplan–Meier estimates for time to clinical worsening, defined as the combined endpoint of death, hospitalisation due to pulmonary arterial hypertension (PAH) complications, use of epoprostenol or prostacyclin analogues for worsening of PAH, lung transplantation, or discontinuation due to worsening of PAH. The Kaplan–Meier estimate was 88% (95% CI 79–97%) at week 16, and 68% (95% CI 55–82%) at week 48.

Summary statistics on clinical worsening are shown in fig 2. The Kaplan–Meier estimate for the absence of clinical worsening was 88% at week 16 and 68% at week 48. Interestingly, there was no clinical worsening among the five patients with SLE at week 48 whereas the estimate for the absence of clinical worsening was 75%, 67%, and 61% for the patients with diffuse SSc, MCTD, and limited SSc, respectively. One patient received intravenous epoprostenol for 10 days during the course of the study.

### Survival

**Figure 3:**
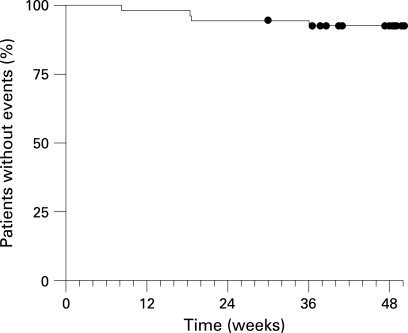
Kaplan–Meier estimates of survival (deaths occurring after treatment discontinuation for an adverse event were counted as an event). The Kaplan–Meier estimate was 92% (95% CI 85–100%) at week 48.

The Kaplan–Meier estimates for the observed survival are presented in fig 3. Survival was 92% at week 48. Four deaths were reported during the study: (1) sudden death, (2) staphylococcal sepsis as a result of ischemic colitis, (3) hyponatremia as a complication of high dose diuretics, including furosemide, and (4) exacerbated dyspnoea from worsening pulmonary fibrosis. These four patients had been diagnosed with limited SSc (n = 2), diffuse SSc (n = 1), and MCTD (n = 1). No patient with SLE died during the study.

### SF-36 and HAQ/VAS scores

The SF-36 domain scores decreased minimally from baseline to week 48. The self-evaluated health transition item at week 48 showed slightly more patients reporting improvement than patients reporting deterioration, in contrast to the baseline status. Accordingly, change in mean (SEM) value for this item was –0.83 (0.22) (95% CI –1.27 to –0.39) (table 2). The HAQ scores presented an overall increase at week 48 (table 3). The disability index was 1.17 (0.11) at baseline and tended to increase at week 48 (0.22 (0.11)). A similar mean increase was reported for all VAS scores at week 48, except for the lung score, which was stable on average (table 3).

**Table 2:** Changes from baseline to week 48 in the Short Form Health Survey (SF-36) domain scores and health transition item

	Baseline	Week 48	Change	95% CI
Domain scores (norm-based)				
Physical functioning*	28.76 (1.24)	27.72 (1.48)	–1.04 (1.37)	–3.79 to 1.71
Role, physical*	27.47 (2.15)	26.19 (1.89)	–1.28 (2.25)	–5.81 to 3.25
Pain	43.00 (1.55)	41.92 (1.74)	–1.08 (1.60)	–4.29 to 2.14
General health perception*	33.89 (1.24)	32.42 (1.24)	–1.47 (1.35)	–4.19 to 1.25
Vitality*	40.63 (1.41)	40.15 (1.47)	–0.49 (1.32)	–3.15 to 2.17
Social functioning	39.67 (1.81)	38.51 (2.13)	–1.16 (2.26)	–5.70 to 3.38
Role, emotional†	32.73 (3.03)	30.65 (3.10)	–2.07 (3.65)	–9.43 to 5.29
Mental health*	45.11 (1.65)	42.94 (1.98)	–2.17 (1.63)	–5.45 to 1.11
Health transition item	3.81 (0.15)	2.98 (0.18)	–0.83 (0.22)	–1.27 to –0.39
Health transition item (n)				
1: Much better	3	4		
2: Somewhat better	2	17		
3: About the same	6	9		
4: Somewhat worse	26	10		
5: Much worse	10	7		

Values are mean (SEM). Four patients were discontinued because of pulmonary arterial hypertension (PAH) worsening/death and were assigned the worst value observed over the analysis set at week 48, as per protocol. The health transition item is reporting comparison to 1 year before. A decrease (negative change) in a domain score corresponds to deterioration.

n = 47, *n = 46, †n = 45.

**Table 3:** Changes from baseline to week 48 in the Health Assessment Questionnaire (HAQ) and visual analogue scale (VAS) scores on a 0–3 scale

	Baseline	Week 48	Change	95% CI	
HAQ scores					
Dressing	1.15 (0.16)	1.49 (0.19)	0.34 (0.16)	0.01 to 0.67	
Arising	0.77 (0.13)	1.09 (0.17)	0.32 (0.20)	–0.08 to 0.72	
Eating	0.81 (0.13)	0.98 (0.17)	0.17 (0.16)	–0.16 to 0.50	
Walking	1.32 (0.14)	1.60 (0.18)	0.28 (0.16)	–0.05 to 0.60	
Hygiene	0.98 (0.15)	1.30 (0.19)	0.32 (0.18)	–0.04 to 0.68	
Reach	1.28 (0.16)	1.62 (0.18)	0.34 (0.16)	0.01 to 0.67	
Grip	1.15 (0.17)	1.28 (0.18)	0.13 (0.17)	–0.22 to 0.48	
Activity	1.94 (0.14)	1.98 (0.17)	0.04 (0.14)	–0.23 to 0.32	
HAQ disability index	1.17 (0.11)	1.39 (0.14)	0.22 (0.11)	–0.01 to 0.44	
VAS scores					
Pain	0.87 (0.11)	1.06 (0.13)	0.19 (0.16)	–0.13 to 0.51	
Gastrointestinal	0.44 (0.10)	0.67 (0.11)	0.23 (0.11)	0.0 to 0.46	
Lung	1.71 (0.11)	1.70 (0.14)	–0.02 (0.14)	–0.29 to 0.26	
Vascular	1.11 (0.13)	1.32 (0.15)	0.21 (0.19)	–0.18 to 0.60	
Digital ulcer	0.58 (0.12)	0.86 (0.16)	0.28 (0.16)	–0.03 to 0.59	
Disease	1.39 (0.13)	1.56 (0.14)	0.17 (0.13)	–0.10 to 0.44	

Values are mean (SEM), n = 47. Five patients were discontinued because of pulmonary arterial hypertension (PAH) worsening/death and were assigned the worst value observed over the analysis set at week 48, as per protocol. A negative change corresponds to an improvement of the HAQ and VAS scores.

### Safety

The most frequent adverse events (% patients) observed during the study were peripheral oedema (17%), liver enzyme elevations (17%, of which 11% were specified as aminotransferase increases), diarrhoea (13%), exacerbated dyspnoea (13%), and nausea (13%).

At least one serious adverse event was reported in 45% of patients (37 events in 24 patients). The most frequent serious adverse events were exacerbated dyspnoea (8%) and pneumonia (8%). All serious adverse events were judged unrelated to study medication by the investigators.

Overall, 14 patients (26%) discontinued study medication because of an adverse event or serious adverse event. Discontinuations most often involved exacerbated dyspnoea (6%), general physical health deterioration (6%), and liver enzyme increase (6%).

## DISCUSSION

This is the first large multi-centre prospective single-arm study of survival and quality of life in PAH–CTD confirmed by right heart catheterisation. The results represent an important source of data on survival and quality of life in patients with PAH–CTD treated with bosentan.

The survival estimate of 92% with only slight alteration in the patients’ quality of life can be considered as a positive outcome in PAH–CTD, a progressive disease with very poor outcome in the absence of specific therapy. Treatment of PAH with bosentan was indicated in the patients included in this study. Hence, a placebo therapy for 48 weeks would have been unethical. The absence of a placebo group may be considered a limitation of the study, with concern that some of the improvements were due to “placebo” effect, rather than drug efficacy. However, non-subjective parameters, such as survival, cannot be explained by a placebo effect. Previously reported placebo-controlled PAH studies of bosentan or other PAH-specific treatments have not shown any clinically relevant improvements in placebo groups, in fact, they have generally shown a decline.^13^ ^14^ ^20^ ^21^ ^35^^–^37 In addition, a historical comparison shows that the observed estimate for absence of clinical worsening at week 16 is similar to the rates reported for the PAH–CTD patients treated with bosentan in the placebo-controlled BREATHE-1 study.^21^

The survival results of the study are in line with published data in patients treated with bosentan. In particular, the observed survival rate of 92% is comparable with the 1-year survival of 86% reported by Denton *et al*^23^ in a subgroup analysis of 64 patients with PAH–CTD (SSc, SLE, MCTD), who were enrolled in the two bosentan placebo-controlled PAH trials and the open-label extensions. Similarly, Williams *et al*^38^ reported a survival at 1 year of 81% in a cohort of 45 patients with PAH related to SSc in class III–IV who were treated with bosentan as first line therapy. In contrast, the survival reported by these authors for the historical control (47 patients treated with basic therapy with (n = 27) or without (n = 20) prostanoids) was 68%.^38^ Data on the effects of injectable prostanoids and sildenafil on haemodynamics, exercise capacity and symptoms in PAH associated with connective tissue disease have been presented.^39^ ^40^ However, their effect has not been studied in a specific long-term cohort in this patient population.^41^ ^42^

The results of the study also suggest that bosentan improved or stabilised the clinical status of most patients with PAH–CTD. In 84% of patients, the WHO functional class improved (27%), or remained unchanged (57%) at week 48.

The self-evaluated SF-36 health transition item showed more improvements than deteriorations in overall health perception at the end of the study, as well as an improvement in this overall perception compared to the year preceding the study. The decrease in SF-36 domain scores was lower than that generally considered as the minimal clinically important difference.^43^ The apparent discrepancy between the encouraging results of the SF-36 health transition item and the decrease in the domain scores can be explained by the different nature of these measures. Whereas the health transition item is a general and relative (compared to 1 year ago) measure, the domain scores are specific and absolute (providing a value at the date of assessment) measures.

Patients included in the current study had moderate-to-severe baseline disabilities^44^ (HAQ disability index  = 1.17 (0.11)). At week 48, the observed mean increases in the HAQ disability index (+0.22, 95% CI –0.01 to 0.44) reached the minimal clinical important difference value reported for patients with SSc (0.10–0.22)^45^ ^46^ and the VAS scores increased by less than 0.3 units, except for the lung score which was stable. Interpretation of these findings is limited by the absence of a control group. In a different cohort of SSc patients without PAH followed for a mean of 1.8 years, the HAQ disability index increased by 0.4 units and VAS scores by 0.5–0.6 despite state of the art therapy.^47^ Hence, our findings suggest that bosentan PAH therapy was specifically associated with a stabilisation of the lung score despite other coexisting CTD respiratory manifestations, but, as expected, had less impact on other organ-related quality of life indicators.

As for any instrument, the HAQ/VAS and SF-36 tools have well known limitations: the patients’ judgments about the extent of their disabilities may show marked individual variation,^48^ the HAQ/VAS does not capture the psychological distress felt by patients with PAH and/or CTDs,^49^ ^50^ and neither instrument is specific for PAH. Consequently, they may have limited sensitivity in detecting changes in quality of life resulting from PAH treatment over time.^51^ New instruments, which were not available at the start of this study, such as the Cambridge Pulmonary Hypertension Outcome Review (CAMPHOR)^51^ have been developed from qualitative, unstructured interviews with PAH patients and are expected to provide a more accurate assessment of the impact of PAH on quality of life, when validated translations permit their use in international studies.

The minimum TLC for inclusion in our study was 50% of predicted. Low TLC may reflect interstitial lung disease or chest wall restriction or other cause for low lung volumes such as pleural disease. There was evidence of lung fibrosis in some cases but our experience and previously published data^52^ ^53^ suggest that outcome is especially poor in CTD cases, particularly SSc, in which there is RHC proven pulmonary hypertension together with lung fibrosis. Hence, the inclusion of these patients would be more likely to have had a negative impact on the overall prognosis in the cohort.

In conclusion, our study suggests that patients with PAH–CTD can be treated with bosentan in centres having PAH in addition to CTD expertise and that bosentan is effective for the treatment of this patient population. Our results suggest that bosentan improves or stabilises the clinical status in the majority of PAH–CTD patients and, most importantly, has a positive impact on survival. In contrast to the observed changes of SF-36 and HAQ scores not related to PAH, the HAQ lung VAS score stabilised and the SF-36 health transition item improved. Altogether, these results are consistent with sustained benefit in this patient population, which is usually characterised by a high morbidity and mortality.
